# Synthetic Peptides as Therapeutic Agents: Lessons Learned From Evolutionary Ancient Peptides and Their Transit Across Blood-Brain Barriers

**DOI:** 10.3389/fendo.2019.00730

**Published:** 2019-11-12

**Authors:** David A. Lovejoy, David W. Hogg, Thomas L. Dodsworth, Fernando R. Jurado, Casey C. Read, Andrea L. D'Aquila, Dalia Barsyte-Lovejoy

**Affiliations:** ^1^Department of Cell and Systems Biology, University of Toronto, Toronto, ON, Canada; ^2^Protagenic Therapeutics Inc., New York, NY, United States; ^3^Department of Pediatrics, University of Alabama, Birmingham, AL, United States; ^4^Structural Genomics Consortium, University of Toronto, Toronto, ON, Canada

**Keywords:** stress, latrophilin, receptor-ligand interaction, neuroplasticity, blood-brain barrier, G-protein coupled receptors, secretin, CRF

## Abstract

Peptides play a major role in the transmission of information to and from the central nervous system. However, because of their structural complexity, the development of pharmacological peptide-based therapeutics has been challenged by the lack of understanding of endogenous peptide evolution. The teneurin C-terminal associated peptides (TCAP) possess many of the required attributes of a practical peptide therapeutic. TCAPs, associated with the teneurin transmembrane proteins that bind to the latrophilins, members of the Adhesion family of G-protein-coupled receptors (GPCR). Together, this ligand-receptor unit plays an integral role in synaptogenesis, neurological development, and maintenance, and is present in most metazoans. TCAP has structural similarity to corticotropin-releasing factor (CRF), and related peptides, such as calcitonin and the secretin-based peptides and inhibits the (CRF)-associated stress response. Latrophilins are structurally related to the secretin family of GPCRs. TCAP is a soluble peptide that crosses the blood-brain barrier and regulates glucose transport into the brain. We posit that TCAP represents a phylogenetically older peptide system that evolved before the origin of the CRF-calcitonin-secretin clade of peptides and plays a fundamental role in the regulation of cell-to-cell energy homeostasis. Moreover, it may act as a phylogenetically older peptide system that evolved as a natural antagonist to the CRF-mediated stress response. Thus, TCAP's actions on the CNS may provide new insights into the development of peptide therapeutics for the treatment of CNS disorders.

## Introduction

The central nervous system (CNS) communicates information to the peripheral tissues primarily by neurotransmitter-mediated modulation of tonic and phasic ionic conductance among cells. Because of the large amount of information that can be encoded by this modulation, only a handful of small molecule neurotransmitters are required. However, secondarily, the CNS employs neurosecretory signals, mostly in the form of amphiphilic peptides that are frequency- and amplitude-modulated to regulate the activity of proximal endocrine organs and tissues. Both transmission processes signal to the periphery and convey sensory information from the integration of in-coming external and internal organismal signals. As is the case with all transmission-reception systems, the CNS necessarily obtains feedback information regarding the physiological state of the peripheral tissues and organs. This receptive information occurs primarily via non-neural signals.

**Figure 1:**
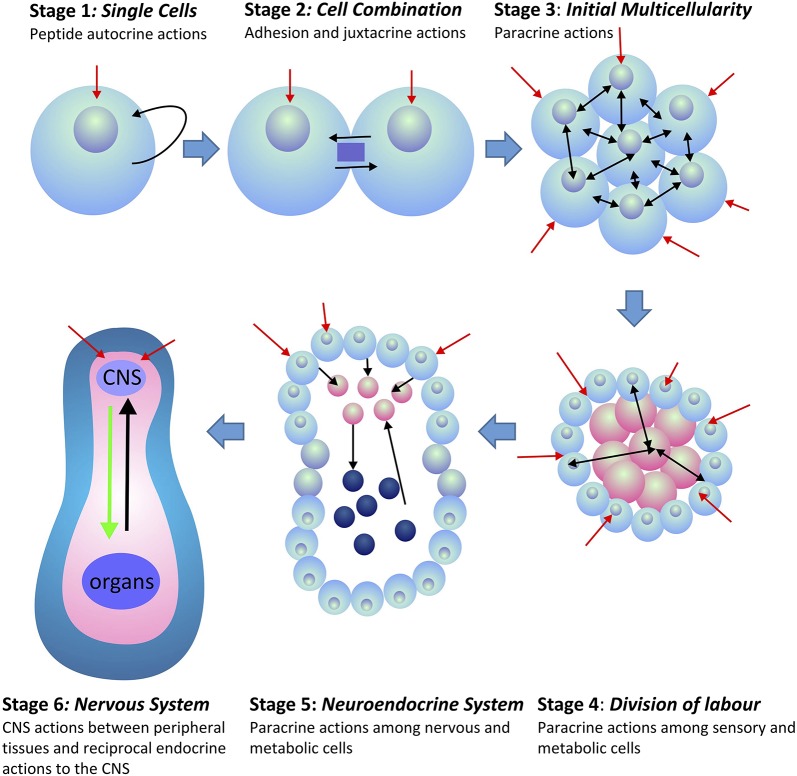
Evolution of peptide signaling and information transfer in metazoans. Red arrows indicate external and environmental sensory information transfer into the organism. Black arrows indicate internal information transfer among cells and tissues. Stages 4–6 indicate the separation of tissues between those that receive external sensory information (blue) and those that are associated with the internal homeostatic interactions of the organism (pink). In Stage 6, internal peripheral signals from internal organs and tissues are indicated in black, whereas, CNS outflow to the peripheral organs and tissues is indicated in green.

This CNS outflow of sensory information to the periphery combined with the counter-flow of information on the status of the peripheral tissues back to the CNS forms the basis of homeostatic regulation in all multicellular animals (metazoans). This system had to be functional in order for the first metazoans to evolve. Peripheral tissues, being non-neural in nature, can only provide feedback information to the brain by the release of secreted compounds that do not allow tonic or phasic information encoding. Moreover, given the number of specialized tissues that exist amongst the peripheral organs, their signal individuality can only be encoded by the suite of chemical compounds released into the interstitial space and vascular systems. Such signals provided coordination amongst peripheral tissues, and later became integrated into CNS communication. This integration occurred over much time that incorporated a number of evolutionary stages. The development of multicellular organisms led to a division of labor among cells. Further development led to the formation of functionally specific tissues and organs. Depending upon the available genome and associated gene expression in these tissues, the expressed secretory peptides had the potential to provide specific tissue and organ information back to the brain (Figure 1). However, because peripheral tissues evolved from shared common tissues and organs, and due to their associated genome, homeostatic information had to be encoded within the identity and structure of the secreted compounds. Peptides were particularly appropriate for this task as unique information could be encoded not only in frequency and amplitude modulation, but also within their primary amino acid sequences, and their subsequent secondary and tertiary structures [see (1) for discussion].

Reciprocal information exchange between the CNS and the periphery occurred before the evolution of CNS-vascular barriers. This was likely an evolutionary response to protect the integrity of intra-organismal information transmission from potential disruption of the homeostatic state by extra-organismal signals. As the blood brain barrier (BBB) evolved in the Metazoa, changes in its structure were evolutionarily selected to protect the organism from potentially noxious external signals while protecting incoming internal signals. For these reasons, the ability of synthetic therapeutic compounds, to transit across the BBB has been problematic, as these structures are unique to metazoan evolution over the last 600 million years, and therefore, frequently do not possess biological structures amenable to crossing the BBB. Natural peptides that evolved before the formation of the BBB co-evolved along with the development of the BBB, thus as a result, these peptides possessed the structural primary amino acid motifs and the subsequent secondary structures that were critical as essential recognizable regions by the early receptors and transporters that allowed transit across the BBB. Although numerous peptides do, in fact, cross the BBB, the lack of success of numerous synthetic pharmaceutical-based peptides is due, in part, to the focus on receptor-ligand binding *in vitro* as opposed to the requirements of a peptide to be soluble in different tissues, resistant to vascular-, and tissue-based peptidases, possess an extended resistency time in the target tissues, yet still be excreted by the organism.

However, recently, there has been more attention paid to the role of peptide-based therapeutics in the Pharma industry. Unfortunately, the inability of many of these synthetic peptides, novel to the biology of their target organisms (i.e., mammals, humans) to transit into the CNS has led to the misunderstanding that peptides, *per se*, do not enter CNS from the vasculature. Recent studies have identified numerous peptides that cross these vascular CNS barriers and have provided a rationale for this selective mechanism. The teneurin C-Terminal associated peptides (TCAP) evolved before the advent of various brain barriers, and is structurally related to many of the peptides that do cross the BBB. We posit that this is the reason for the success of TCAP-related peptides to transit among neural and peripheral tissues to achieve potent physiological and behavioral actions.

## Peptides and the Blood Brain Barriers

Historically, it was thought that peptides could not pass through the BBB. This early understanding was based on both the lack of understanding of the physiology of the BBB and the available technology to examine peptidergic transmission across the BBB (2, 3). However, by the last quarter of the twentieth century, Bloom (4) established that gastrointestinal peptides communicated with the brain, therefore, across the BBB, as part of a feedback mechanism to regulate organismal homeostasis. This was a major step forward in the understanding of inter-organ physiology and its relationship to the brain. The basic concept underlying this mechanism is that while the central nervous system (CNS) can communicate with non-neural tissues by nervous and neuroendocrine output, reciprocal efferent signals from these tissues must necessarily employ endocrine factors.

Since then, numerous peptides have been shown to cross the BBB by a combination of receptor and transporter mechanisms in specific vascular regions of the brain [see (2) for review]. However, despite the large number of studies confirming the transit of peptides across the BBB, numerous peptides do not cross. Because the BBB evolved to protect the brain from exogenous and potentially toxic compounds, yet needed to provide a portal to allow communication with endocrine signals from peripheral tissues, endogenous peptides that play a major role in homeostatic regulation are more likely to cross the BBB. A number of peptides such as insulin, leptin, ghrelin, and members of the secretin superfamily of peptides such as pituitary adenylate cyclase-activating polypeptide (PACAP), vasoactive intestinal peptide (VIP), and urocortin can cross the BBB (see below).

### Vascular and Neural Access of Peptides to the CNS

Peptide action on the brain is regulated by a number of pharmacokinetic aspects that ultimately affect residency time in tissues. Residency time of a peripherally injected peptide in the brain is based on several factors. First, the clearance rate, including peptide degradation by plasma and endothelial associated peptidases, and elimination via the urinary system; second, transport into non-neural tissues; third, transport kinetics in the brain via the various neural blood barriers; and finally, efflux out of the brain.

Under natural conditions, most terrestrial vertebrates have three major routes that allow the intake of bioactive peptides from exogenous sources, including environmental (xenobiotics arising from natural products and anthropomorphic activities), nutrients (food) and pharmaceuticals (administered drugs). The best studied of these routes is by ingestion. However, this method is not practical due to the high level of degradation that occurs in the gastrointestinal (GI) tract. Even those peptides that do survive and are taken up across the gut are further degraded by endothelial peptidases, and/or are eliminated by the kidney. Typically, most peptides have a half-life in the blood in the order of minutes and generally do not accumulate in the tissues (2). Thus, for the introduction of peptides into an organism that will ultimately enter the brain, an oral route is rarely practical. Although peptide ingestion by the gustatory route may provide limited access of some bioactive peptides that are relatively resistant to GI-associated degradation, this physiological system ultimately evolved to obtain nutrients that are safe to the organism. This complex transitory route is impractical for the learning of social interactive behaviors, as in many cases, the threat or invitation may be over by the time the low amounts of the remaining intact bioactive compound has been perceived by the brain. Thus, for these reasons, other routes that provide a more direct route to the sensory integration regions of the CNS have evolved.

Over the last decade, peptide administration via nasal or oral mucosa administration has received greater attention. There are a number of reasons for this. Both the olfactory and vomeronasal (VNO) organs evolved specifically to take in chemical cues from the environment in an efficient manner and allow the organism to make rapid decisions that affect survival. The two sensory systems differ in that the olfactory organ is predisposed to sample volatile chemical signals, whereas the VNO is more sensitive to non-soluble and/or non-volatile compounds [see (1) for discussion]. Depending upon the species, these organ systems may be separate or integrated. Importantly, both organs are associated with a vascular network that is closely associated with the CNS, allowing for greater concentrations of the active compound to reach the CNS. The VNO is present in most vertebrates but possesses a number of specialized adaptations. Depending upon the species, access to the vasculature may occur via the dorsal oral mucosa or via the nasal epithelium. In those vertebrates that lack a VNO, evidence indicates that this a derived condition associated those lineages (5–7). Although this organ is well-developed in rodents, in humans, the VNO has been evolutionarily modified. The vomeronasal epithelium develops early in fetal life and is the embryonic tissue source of gonadotropin-releasing hormone (GnRH) neurons that migrate into the telecephalon to ultimately regulate the neuroendocrine aspects of the reproductive system (8). In the second half of pregnancy, the sensory aspects of the vomeronasal epithelium (VNE) degrades due, in part, to mutations of genes specific to VNO function (9). The adult human VNO possesses a number of features similar to that of the fetal VNE and that of non-human vertebrates, although it appears to have lost its sensory capacity (5–7, 10). However, in adult humans, there are a number of connections of VNO cells with the underlying capillaries, indicating that the VNO has evolved to take on a more endocrine function (11). Although the role of VNO with respect to peptide uptake in humans is equivocal, the actions of the main olfactory epithelium are less so. The sensory neurons of the olfactory epithelium extend their dendrites into the nasal cavity thus allowing these sensory neurons to come into direct contact with the external environment (12). As a result, olfactory signals can directly interact with the CNS.

Arguably, the least understood of these delivery methods are those that utilize the oral mucosa. In humans, these include the buccal mucosa (lining of the cheek), the sublingual mucosa (floor of mouth and underside of the tongue), and the gingival mucosa (associated with the teeth and aspects of the jaws). Transport of proteins and peptides across the oral mucosa occurs primarily by passive diffusion and avoids GI degradation via bypass of the initial hepatic metabolic processes (13). Transit of peptides through this route into the brain can be relatively efficient in comparison to other methods of administration, however, depending upon the peptide structure and solubility, can be limited by the comparatively small epithelial region and the difficulties in maintaining a constant delivery concentration (14).

### CNS Blood Barriers

Although the vascular system of the olfactory, vomeronasal organs and oral mucosa indicates that peptides taken up via these routes gain a greater access to the CNS, they still need to navigate the various blood barriers of the CNS. There are three main barriers that control the molecular transport between the blood and the neural tissues: the blood brain barrier (BBB), which is formed by the cerebral vasculature epithelium between the blood and the brain's interstitial fluid; the choroid plexus epithelium between the blood and ventricular cerebrospinal fluid (CSF) barrier (2, 15, 16); and the arachnoid epithelium between the blood and the ventricular CSF. However, it is the BBB that has the greatest control of the proximal environment of brain cells (3).

There are two basic mechanisms by which a peripheral peptide can affect neuronal function. Peptides may be transported across the BBB via passive diffusion or saturable transport (2), resulting in activation of receptors at the cerebral vasculature level (17). Although it has been suggested that almost 100% of large molecules cannot cross the BBB (18), this number is misleading (3). Only about 0.05% of injected insulin reaches the brain (19). Previous studies lacked the technology and sensitivity to detect low amounts of the peptide in the brain. In the case of morphine, although it is not a peptide, about 0.02% of the injected dose can be detected in the brain (20). Thus, it is not appropriate to compare peptide transit across the BBB directly with other small bioactive molecules. It is likewise not reasonable to expect that peptide transit in the brain will occur via the circumventricular organs (CVOs). There is a 5,000-7,500-fold difference between the surface area of the CVOs as opposed to that of the BBB (21, 22). Moreover, the morphology of the capillaries associated with the CVOs do not allow significant penetration of peptides into the brain (16). As an example of this relationship, the transit of interleukin 1 alpha (IL-1α) across the CVOs accounts for <5% of the peptide that enters the brain (2, 23).

Taken together, the previous studies of peptide transit across the various BBBs indicate several essential attributes of these barriers. Most importantly, this barrier evolved to ensure interactive transit between the peripheral tissues and the brain to ensure homeostatic communication among all tissues and organs of the organism. Secondarily, the specificity of compounds that could transit the BBB was likely based on the suite of soluble proteinaceous and metabolic compounds as defined by the genomes of the species. Given this evolutionary scenario, it explains why the majority of natural xenobiotic compounds and novel artificially-derived compounds do not readily cross the BBB. In other words, after over 600 million years of selective evolution of the BBB, it is unlikely that a novel non-natural synthetic peptides that does not encompass the structural attributes for membrane transit, refractile to peptidases, yet still possesses a high affinity (Kd < 1 nM) for its target in the brain, will be successful. Peptide therapeutics based on the structure of natural peptides that possess a long evolutionary history are candidates for therapeutics that could be used for the treatment of mood disorders. The teneurin C-terminal associated peptide (TCAP) possesses the attributes that make it an excellent candidate for the treatment of mood disorders.

## Discovery of the Teneurins and Their Receptors

The existence of the teneurins was reported independently in 1994 by two separate laboratories (24–26). Within a few years, the teneurin family was acknowledged as a type-II transmembrane protein that was highly expressed in the central nervous system (CNS) of almost all metazoans. The teneurins are complex multifunctional proteins consisting of numerous functional domains translated from a gene consisting of 20 to over 30 exons (27, 28) spanning over 600,000 bases in the genome. There are generally 4 teneurin paralogues found amongst chordates, thereby conforming to the 2R hypothesis that theorizes two major genome duplication events in the Chordata (29, 30). In contrast, there is typically only a single gene found in invertebrates with the exception of the Insecta, which possess two paralogues. Initial studies of the teneurins indicated a major role in neural development included cell adhesion, axonal pathfinding and cell proliferation (27, 28). The teneurins have now emerged as critical genes required for normal CNS function and maintenance.

For the first two decades following the discovery of the teneurins, the receptor mechanism was not clear. Initial studies indicated that the teneurins could homo-and hetero-dimerize to achieve activation (31–35). This teneurin activation stimulates the cleavage of its intracellular domain that leads to translocation and activation of the nuclear transcription factor zic-1 (31, 33). Although this may indeed be the case in some situations, new studies in the last decade have suggested an alternative hypothesis that the teneurins interact with a family of Adhesion family G-protein coupled receptor (GPCR) known as the latrophilins (LPHN). The LPHNs comprise a family of three paralogous receptors (36, 37). LPHN is also known as calcium-independent receptor for latrotoxin (CIRL), reflecting its binding capacity for α-latrotoxin (α-LTX) (38, 39). Their structure contains a long extracellular portion comprised of a lectin-like domain, an olfactomedin-like domain, a hormone-binding domain and a GPCR autoproteolysis inducing (GAIN) domain, which includes a GPCR proteolytic site [GPS; (39)]. This is followed by the seven-transmembrane domain that defines all GPCRs and, finally, a C-terminal intracellular tail. The LPHNs were first discovered in the search for the calcium-independent receptor of α-LTX, the principle vertebrate toxic component in the venom of the black widow spider (genus *Lactrodectus*) (38). Although α-LTX was initially shown to bind to the neurexins in an interaction that is calcium-dependent (40, 41); it also caused downstream effects in calcium-absent conditions, indicating another potential receptor mechanism at play (42). Davletov et al. (38) were the first to purify LPHN from detergent-solubilized bovine brain membranes and established that it bound α-LTX *in vitro* with high affinity in the absence of calcium, indicating a receptor-ligand interaction between the two molecules. This was further established via over-expression expression of LPHN in chromaffin cells, which resulted in increased cell sensitivity to α-LTX (39).

After their initial discovery, the three LPHN isoforms were classified as members of the Secretin GPCR family, as their hormone binding domains showed high sequence similarity to the signature hormone binding domains of the Secretin GPCRs (43). These receptors have since been re-classified to the Adhesion GPCR family due to their long extracellular domains containing adhesion motifs and associated adhesion functions (44, 45). Recent phylogenetic analyses indicate that the Adhesion GPCR family is ancestral to the Secretin GPCR family, and that the Secretin GPCRs inherited their hormone binding domain from the Adhesion GPCRs (37, 46, 47). As this domain is critical to Secretin ligand binding, the ligands of the Adhesion GPCRs may have also been the progenitors to the Secretin GPCR ligands.

## Discovery and Characterization of the Teneurin C-terminal Associated Peptides

Qian et al. (48) identified a clone from a rainbow trout hypothalamic cDNA library representing an ortholog of teneurin-3. This led to the discovery of a peptide-like sequence encoded at the carboxy-terminus in the last exon of the rainbow trout teneurin-3 gene. Because this sequence was annotated as part of the teneurin gene, this region was termed Teneurin C-terminal-associated peptide (TCAP)-3. TCAP-1,−2, and−4 were subsequently identified following *in silico* analyses of the available teneurin-1,−2, and−4 sequences, respectively (49). The TCAPs are approximately the same size as both CRF and its direct paralogues, urotensin-I (UI), and urocortin (Ucn), ranging from 40 to 41 residues in length. The TCAP and CRF families of peptides possess about 30% sequence similarity among homologous replacements (28, 50, 51). In addition, the TCAPs possess the cleavage motifs similar to CRF and related peptides (52). This primary structure similarity suggested that the TCAP family was distantly related to the CRF peptide families, and that they may share a common evolutionary origin (53, 54).

**Figure 2:**
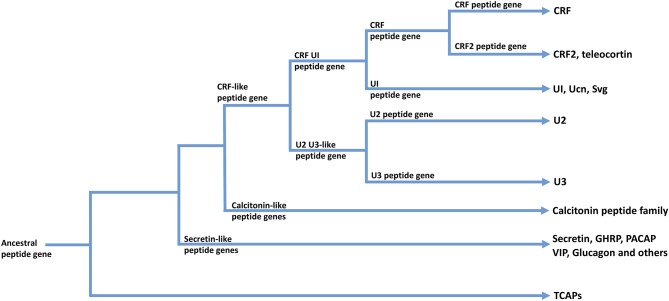
A possible scheme for the phylogenetic relationships among TCAP-, CRF-, Calcitonin-, and Secretin-associated peptide families. Arrows indicate peptide families found in extant organisms.

The CRF family of peptides belong to the Secretin family of peptides (28, 50, 54, 55). The CRF family consists of four to five paralogous peptides that mediate the stress response and regulate stress-associated energy metabolism. CRF is fundamentally responsible for regulating the hypothalamic-pituitary-adrenal (HPA) axis and coordinating the peripheral endocrine response to stress (56, 57). In vertebrates, the CRF family of ligands is highly conserved and integrated into a number of diverse physiological systems. This indicates significant selection pressures to maintain the CRF ligand-receptor signaling system due to the fundamental physiological roles it plays (58). The vertebrate CRF ligand family is comprised of two paralogous lineages: CRF and its direct paralogues as one lineage; and urocortin 2 and 3 that are included within a second paralogous lineage. The first paralogous lineage includes CRF and CRF2 [teleocortin; (55, 59–61)]; and a second lineage that includes mammalian urocortin (Ucn), amphibian sauvagine (Svg), and fish urotensin-I (UI) (62, 63). A second paralogous lineage to the CRF and UI family lineage includes urocortin 2 (Ucn2) and urocortin 3 (Ucn3) (64–66) (see Figure 2). Within invertebrates, the diuretic hormones (DHs) are orthologous to the CRF family of peptides, and are predominantly involved in osmoregulation and diuresis in insects (63, 67, 68). The secretin-related peptides are widely expressed in deuterostomes and are also present to a limited degree in protostomes, however there are no clear data among lineages that evolved before the evolutionary divergence between these two lineages (55).

In contrast, evidence indicates that the teneurin-TCAP system predates the CRF-secretin peptide family by a few hundred million years. Comparative genomic and protein analyses support the theory that the teneurin/TCAP complex originated in metazoans through a horizontal gene transfer event from prokaryotes to a single-celled metazoan ancestor, likely a choanoflagellate (69–72). Prokaryotes contain unique proteinaceous polypeptide toxins (PPTs), which possess several characteristics similar to those of metazoan teneurins, including a type-II orientation and three main domains: an intracellular domain involved in secretion or cell signaling, a central domain involved in adhesion, and a C-terminal domain containing a toxin payload that could be released into neighboring cells (69, 72). The TCAP portion of teneurin, specifically, has high amino acid sequence similarity to the glycine-histidine-histidine (GHH) clade of the PPT C-terminal toxin domains (72). Additionally, the teneurins are the only metazoan genes to contain YD repeats, a motif common in certain aquatic bacteria and with similarity to bacterial RHS elements (70, 73). The recently resolved structures of chicken and human teneurin-2 and mouse teneurin-3 also show a striking similarity to that of bacterial Tc-toxins, with a cylindrical β-barrel domain encompassing a toxin-like C-terminal region corresponding to the TCAP region (74, 75). However, the teneurin extracellular domain itself also contains eight epidermal growth factor (EGF)-like repeats, which are a hallmark of the metazoan genes (71). Thus, the current theory regarding the evolution of the teneurins posits that a PPT was inherited by a choanoflagellate from a prokaryote via horizontal gene transfer, and subsequently became associated with an EGF repeat domain, resulting in the formation and expression of a proto-teneurin gene containing an extended extracellular region and a bioactive C-terminal domain (69, 72). In bacterial PPT toxins, the encapsulated carboxy region containing the toxic payload (i.e., GHH and TCAP) is cleaved and released by proteases within the barrel domain (76). Although the function of the barrel domain in teneurins is yet to be determined, it may act on one of several known putative cleavage sites upstream to TCAP to allow for its release (75). Tucker (73) has postulated that the introduction of the teneurin protogenes into the choanoflagellates may have acted to increase the entrapment of their algae prey, thus linking the teneurins with nutrient acquisition and energy metabolism.

Although the dynamics of the interaction between Teneurin and TCAP as LPHN ligands was initially unclear, subsequent studies showed that Teneurin-2 bound with nanomolar affinity to the lectin-domain of LPHN1. Furthermore, a splice variant of C-terminal domain of Teneurin-2, termed LPHN1-associated synaptic surface organizer (Lasso), could also bind to LPHN1 at its C-terminal globular domain with high affinity, and with the implementation of antibodies against each of the ADGRL homologs, they demonstrated that LPHN1 is the primary ligand of Lasso (77). Furthermore, Teneurin-1 and Teneurin-4 also bind LPHN1 (78, 79). Because both the Teneurin paralogues and Lasso bind to LPHN1 with high affinity, it was presumed that TCAP could bind with the LPHN family. Transgenic over-expression of both TCAP-1 and the hormone-binding domain (HBD) of LPHN1 showed that both signals could be detected in immunoprecipitation studies. Moreover, the transfected cells experienced high levels of cytoskeletal rearrangement in the presence of TCAP-1 compared to wild-type cells (80). Together, these studies indicate that the extracellular region of teneurins, including TCAP-1, bind with LPHN1. Importantly however, these studies link TCAP-1 as a peptide to the Secretin family of peptides that bind to a receptor phylogenetically linked to the Secretin family of GPCRs. Given that both the teneurin/TCAP protein ligand and the LPHN receptor are the apparent result of lateral gene transfers from prokaryotes to a single celled metazoan ancestor (72), this suggests that this ligand-pair evolved well before the evolution of the Secretin-like ligands and associated receptor superfamilies.

### TCAP, Secretin Peptides, and Relationship to Energy Metabolism

Further indication that TCAP is evolutionarily ancient and that it is a potential progenitor of secretin-like peptides along with the CRF and CRF-like peptides is supported by a number of physiological studies. Considerable evidence establishes a commonality among the secretin peptide and secretin receptor superfamilies (including CRF and calcitonin) in the regulation of energy metabolism for homeostasis (81–83). Much like the Secretin family of peptides, TCAP has been implicated in the regulation of both cellular and organismal energy metabolism (82, 84, 85). Recently, TCAP-1 has been demonstrated to induce glucose uptake in murine neuronal cells *in vitro* using ^3^H-deoxyglucose (DG) (86). As glucose is the primary energy substrate in the brain, increased neural glucose uptake into rat brain using functional positronic emission tomography with ^18^F-DG indicates that TCAP-1 can regulate the supply component (i.e., glucose) of a cell's energy budget (86). Importantly, *in vitro* studies indicate that TCAP-1-mediated glucose uptake occurs through an insulin-independent pathway. Moreover, phylogeneticaanalyses suggest that TCAP evolution also predates insulin (86–88), this indicates that TCAP may be one of the first signaling peptides to regulate glucose uptake in metazoans.

If the fundamental role of TCAP was to regulate energy metabolism in the earliest metazoan ancestors, then it is possible that that TCAP's earliest functions were to stimulate aerobic metabolism, glucose, and other nutrient importation, and mitochrondrial activity. This mechanism would necessarily be associated with cellular energy homeostasis given its evolutionary history before it was modified by the formation of novel functionally related paralogues arising from gene and genomic expansion by later multicellular organisms. Thus, we hypothesize that TCAPs original cellular role was to regulate cellular energy homeostasis, and that this mechanism has been conserved throughout the Metazoa. This supposition also suggests that the TCAPs should play a role in most metazoan tissues because the peptide evolved before the development of the multicellular animals and the subsequent differentiation of the various tissues and organs. It may also indicate that TCAP could play a greater role in the most highly energetic tissues.

### TCAP and Transit Across Blood Brain Barriers

Assuming that TCAP evolved as a bioactive peptide in the earliest stages of metazoan evolution, then its presence in metazoans predated the development of CNS-vascular barriers. Peptides structurally similar to TCAP [(54, 68); also see above] utilize a number of mechanisms that allows them to transit into neural tissues. CRF is relatively impermeable to the BBB although miniscule amounts do appear to cross (89). Unusually, CRF is transported out of the brain via a saturable transport system that allows peripheral actions (90). Urocortin-1, the direct paralogue of CRF in mammals, can cross the BBB in small amounts, but permeability is enhanced by co-administration of leptin. Urocortin-2, on the other hand, appears to cross by a passive diffusion mechanism (89). VIP, secretin and GLP-1 likely cross also by a non-saturable passive diffusion mechanism (91). In contrast, PACAP-38 enters the brain by a saturable transport system (3).

Likewise, evidence indicates that TCAP-1 also enters the brain, although the exact mechanism has not been determined. However, previous studies of an IV-administered fluorescein isothiocyanate (FITC)-TCAP1 variant were detected in capillaries and fiber tracts of the caudate putamen and alveolar hippocampus, as well as the anterior cingulate cortex, the cingulum and tracts leading to the choroid plexus (92). A number of the capillaries showed concentrations of fluorescence crossing the endothelial layer. Autoradiographic studies, utilizing IV-administered ^125^I-labeled TCAP-1, similarly showed concentration in the caudate putamen, and cingulate cortex as well as regions of the nucleus accumbens, hippocampus and substantia nigra (86). Interestingly, neither study showed labeled TCAP-1 in any of the CVOs. Although these studies indicated that labeled TCAP-1 could be detected in the CNS after IV-administration, relative to the vehicle, FITC-only and ^125^I-only controls, these studies do not confirm that the intact TCAP peptide was present in the labeled tissues as it is possible that peptide fragments bearing the label may be present.

However, the uptake of the intact TCAP into the brain is supported by a number of physiological studies. Peripheral administration of TCAP produces long-lasting actions that are not easily explained by its apparent short residency time in the CNS (49, 93–95). Several studies indicate that TCAP inhibits the long-term actions of CRF in the CNS. Peptides that do reach the CNS face particular challenges. Even among natural endogenous peptides, they are rapidly degraded by the peptidases of the vasculature and are eliminated via the urinary system. Of the small amount of the peptide that actually reaches its intended target in the CNS, it will be typically internalized and likely degraded. Therefore, this system has evolved to accept the smallest amount of the peptide to obtain a long-term action.

TCAP is an example of such a peptide. The biological half-life of IV-administered TCAP is similar to adrenocorticotropic hormone (ACTH) and its plasma presence is typically removed via the kidneys and urinary tract. However, a single SC- administration of 10 nmol/kg can reduce plasma glucose by 40% for almost 1 week. A similar TCAP-1 administration significantly increases ^18^F-deoxyglucose uptake into the brain after 3 days as assessed by functional positronic emission tomography (86). Moreover, *in vitro*, TCAP induces a significant uptake in ^3^H-deoxyglucose in immortalized neurons due, in part, to a migration of glucose transporters to the plasma membrane. Long-term behavioral indications are similarly affected by TCAP. An ICV regimen of 30 pmols once per day over 5 days decreased the rat acoustic startle response (ASR) by 50% after 3 weeks (49). The ASR is typically used as a measure for anxiety. Using a similar dose/time regimen with either ICV or IV administration, TCAP-1 inhibited the CRF-induced reinstatement of cocaine seeking in rats (93–95).

Assuming the short residency time of TCAP on its CNS receptors, which occurs over a period of minutes, its mechanism may induce long-term synaptic plastic actions in order for these complex behaviors to endure. Evidence for neoplastic actions of TCAP in the brain has come from both *in vivo* and *in vitro* studies. *In vitro* studies utilizing primary and immortalized cell culture showed that TCAP was efficacious at regulating neurite and filopodia formation and axon fasciculation (92, 96). *In vivo*, IV-administration of TCAP-1 induced significant increases in dendritic spine density, and modulation of dendritic arborization (51, 97–99). These *in vivo* modifications of neuronal process development were corroborated *in vitro* by TCAP-mediated expression of a number of cytoskeletal protein mRNAs including β-tubulin α-actinin-4 and β-actin (92). Further studies indicate that the regulation of these mRNAs occurred via a TCAP-mediated phosphorylation of stathmin at serine-25 and filamin A at serine-2152 to stimulate actin and tubulin polymerization (96). Taken together, these studies indicate that TCAP-1 has efficacious neuroplastic actions that may provide an explanation for its long-term action on the CNS despite its expected low residency time on the CNS targets.

## TCAP, CRF, and Mood disorders

Teneurin C-terminal associated peptides evolved both before the formation of the CNS and the BBB and as a result of this, it could possess the necessary structural attributes to pass through a number of tissues and membranes. For this reason, the synthetic version of this peptide, when administered exogenously is efficacious at IV, ICV, and SC delivery. Moreover, this peptide possesses structural similarity to the CRF, calcitonin and secretin associated peptides and, likewise, can regulate glucose metabolism *in vivo*. CRF plays a fundamental role in the regulation of stress-associated energy metabolism and has been implicated in the etiology of mood disorders (100). Thus, taken together, these observations indicated that TCAP and related peptides could play a major role in the treatment of mood disorders including depression, anxiety, and post-traumatic-stress disorder, for example.

A number of TCAP-based studies support such a hypothesis. Initial studies using the acoustic startle reflex (ASR) model, a measure of anxiety, showed that animals with an initial strong response to the SRF showed an attenuation of the response when treated with TCAP. On the other hand animals with a low initial ASR response showed an increased response in ASR (49). In a further study, when both high and low activity animals were combined, then TCAP pretreatment of the animals showed about a 50% decrease in the ASR after 3 weeks of treatment (49) indicating long lasting effects of TCAP. Further studies indicated that the attenuation of the anxiety response by TCAP was due, in part, to the inhibition of CRF actions. With respect to ASR, the expected increase in ASR by CRF could be entirely ablated by treatment with TCAP and could modulate elevated plus maze (EPM) and open field (OP) responses (101, 102), Particularly significant among these CRF-associated studies were the ablation studies of CRF-mediated cocaine seeking reinstatement by TCAP where TCAP inhibited cocaine seeking behavior in rats using both ICV and IV administration of TCAP (93–95). Furthermore, TCAP pretreatment *in vivo* in rats reduces CRF-mediated cfos expression in the limbic regions to basal levels (51, 99, 103).

Overall, TCAP is a natural peptide that possesses the key structural elements that can be used to develop new peptide analogs. The early evolution of TCAP could lead to the creation of a number of other related peptides that are essential for the internal regulation of metabolism and behavior. Although understanding the structural and physiological complexity of peptides will take some time to resolve, peptides in general, have the structural complexity and information transmission among tissues and organs that can act as the foundation for the next generation of drugs and may ultimately act to supplant the use of small molecule–based therapeutics which are typically used as front-line therapies.

The neuroanatomical substrates of mood and neuropsychiatric disorders do not corroborate well with the etiology of patients presenting with clinical symptoms of these conditions. The treatment of mood disorders using the current suite of frontline therapies rarely shows an improved prognosis in >50% of patients. Most of these pharmacological therapeutics incorporate small molecules to regulate monoamine and catecholamine neurotransmitters such as serotonin (5-HT), dopamine and norepinephrine. Despite their efficacy, these neurotransmitters only reach a subset of CNS regions. Because the range of neuroanatomical regions associated with each of these disorders is vast, rarely, if ever, can these therapeutics cover all affected regions of the CNS. Because of this, the medical community has resorted to electrical chemical therapies in the form of deep brain stimulation by transcranial electrode implantation (104), electroconvulsive therapy (ECT) (105), and transmagnetic stimulation (TMS) (106) to treat those patients whose conditions have been resistant to frontline pharmacologic therapies. Although the cellular and molecular mechanisms by which these therapies act is not understood, it does indicate that such approaches have a general effect beyond what is typically seen with any set of pharmacological therapeutics. Finding a commonality of the neuropathology amongst the range of mood disorders has been a particular challenge. One such commonality among all mood and psychiatric disorders is energy regulation. Recently, numerous studies have linked affective disorders such as major depression, anxiety, and post-traumatic stress disorders with CNS energy metabolism (107–110).

## Summary and Conclusions

If we posit that homeostatic peptides regulate the synaptic plasticity of key regions of the CNS associated both with energy metabolism and reward- and fear-based learning behavior, then this indicates that bioactive peptides must, therefore, regulate the energy requirements of the associated neurons. TCAP-based peptides are phylogenetically ancient and are critical to the homeostasis of vertebrates. Because they and their receptor evolved in a single celled progenitor of multicellular organisms, they have become evolutionarily ensconced into numerous metabolic functions, notably within the CNS. In summary, the ancestral gene of TCAP evolved before the advent of the metazoans, and appears to have had a primary function in the regulation of energy metabolism. Its early appearance and maintenance in the genomes of extant metazoans indicates that was essential for the evolution of the Metazoa and may have acted as the ancestral gene that led to the evolution of CRF and the secretin family of peptides. Given this situation, the TCAPs may play a major role in energy metabolism of the brain and its associated pathology.

## Author Contributions

DL and DB-L: development and supervision of all research programs described in the paper. DH: senior PDF overseeing role of energy metabolism of TCAP and CRF. AD'A: developed methods for examining TCAPs role with respect to glycolysis and mitochondrial activity. CR: engaged in the direct role of TCAP on mitochondria. AD'A and CR: prepared the initial drafts of the role of TCAP, peptides, and pharmaceutical therapeutics with respect to the role of mitochondria. TD and FJ: prepared the initial drafts on the history of TCAP peptides.

### Conflict of Interest

DL is a co-founder of Protagenic Therapeutics, Inc., a biotechnology company that has a commercial interest in the therapeutic possibilities of the TCAPs. The remaining authors declare that the research was conducted in the absence of any commercial or financial relationships that could be construed as a potential conflict of interest.
